# Content framing role on public sentiment formation for pre-crisis detection on sensitive issue via sentiment analysis and content analysis

**DOI:** 10.1371/journal.pone.0287367

**Published:** 2023-10-18

**Authors:** Nurul Hidayah Watimin, Hasmah Zanuddin, Mohamad Saleeh Rahamad, Elaheh Yadegaridehkordi

**Affiliations:** 1 Department of Media and Communications, Faculty of Arts & Social Sciences, University of Malaya, Kuala Lumpur, Malaysia; 2 Faculty of Information Science and Technology, Universiti Kebangsaan Malaysia, Bangi, Malaysia; Aravind Eye Hospital and Post Graduate Institute of Ophthalmology, INDIA

## Abstract

Social media has been tremendously used worldwide for a variety of purposes. Therefore, engagement activities such as comments have attracted many scholars due its ability to reveal many critical findings, such as the role of users’ sentiment. However, there is a lacuna on how to detect crisis based on users’ sentiment through comments, and for such, we explore framing theory in the study herein to determine users’ sentiment in predicting crisis. Generic content framing theory consists of conflict, economic, human interest, morality, and responsibility attributes frame as independent variables whilst sentiment as dependent variables. Comments from selected Facebook posting case studies were extracted and analysed using sentiment analysis via Application Programme Interface (API) webtool. The comments were then further analysed using content analysis via Positive and Negative Affect Schedule (PANAS) scale and statistically evaluated using SEM-PLS. Model shows that 44.8% of emotion and reactions towards sensitive issue posting are influenced by independent variables. Only economic consequences and responsibility attributes frame had correlation towards emotion and reaction at p<0.05. News reporting on direction towards economic and responsibility attributes sparks negative sentiment, which proves that it can best be described as pre-crisis detection to assist the Royal Malaysian Police and other relevant stakeholders to prevent criminal activities in their respective social media.

## Introduction

Social media digital space provides online two-way communication which has become very important in our daily lives. Two-way communication works with both sender and receiver to acknowledged and understood the message in concern and sends the feedback via the same channel [1]. This effective communication through social media has become a preference amongst users to engage with one another [1]. Previously, with the concept of two-way communication, people utilised social media for social interaction to catch up with family and friends. Nowadays, it has become increasingly important wherein social media has been further implemented beyond its recognition by the society at large in which its usage is also being utilised for commercial purposes [2].

However, as the technology in social media progresses, relevant features therein have also been expanded, in which, within the digital space, there are definite growing numbers of online virtual communities as well as hate groups utilising the said space to share violent, Islamophobic, and racist narratives which attempt to create a hostile virtual environment and eventually lead to real-world incident such as riot [3–5]. It is crucial to analyse these “new” communities by monitoring the activities they conduct because the content they post might have a damaging impact on community cohesion within society. As such, given the nature of their posts, it is therefore crucial to analyse these “new” communities by monitoring their activities and conducts, as it might result in a damaging impact on community cohesion within the society [6]. It is paramount that an online platform provides a very strategic medium for communication in many concerns. [7–11]. It may also extend to issues relating to criminal acts such as religious hatred and racial tension provocation.

There are many Facebook postings that are widely spread and go viral which contents involve criminal acts allegedly for the purpose of serving justice to certain victims [6]. At a glance, it can be considered a positive posting, as it might serve to help those who are innocent. Nonetheless, such posting might also consist of and relate to sensitive issues, which will subsequently outspread and gets viral without noticing that such posting may create sentiment tension that will potentially initiate and result in an unwanted event such as riot [10, 11].

Riot is one of the criminal activities. Referring to the case study, this riot broke out due to the failure of crisis control by monitoring bodies such as the police and the government to ease the provocation of racial sentiment through social media platforms. The riot incident occurred less than 24 hours after the online provocation began. It is clearly shown that the speed of negative sentiment propagation on social media requires a drastic and efficient strategy to control the spread from triggering communication crisis to a more severe crisis, such as riot that can lead to death.

Manifestly, any organisation nowadays has a team to monitor online issues specifically related to their organisation. However, numerous postings created with variety of negative sentiment based on various issues had been posted not daily but every second, therefore there are millions of such postings [12]. So, it is challenging to recognise which issues are the most threatening, as well as the need to respond promptly and instantly. It is also difficult to predict which issue may be detrimental to national unity based on random observation. Thus, this requires the monitoring body to develop guidelines on how to spot pre-crisis in the posting’s comments. For example, the Plaza Low Yat Racist Riot in 2015 [10, 11] and the Racial Riot at Mariamman Temple in 2018, were two recent occurrences of riots in Malaysia that were sparked by social media provocation. Both cases were initially initiated due to the criminal acts, which had been manipulated on the basis of racial provocation by netizens on social media. The uncontrolled spread of racial sentiment has sparked racial riots and been damaging to the national unity.

The riot incident at Low Yat Plaza had resulted in injuries, but what had happened at the Mariamman Temple Riot was even worse, as death was also involved following the incident. According to the news retrieved from Berita Harian’s official website via the hyperlink https://www.bharian.com.my/berita/nasional/2018/12/510544/kronologi-kes-kematian-muhammad-adib, the fireman, Muhammad Adib Muhammad Kassim, who is suspected of having been beaten so badly which had died after a few weeks of intensive treatment in the hospital.

Analysing social media activities, known as engagement activities, is therefore crucial in order to overcome unwanted events on social media. [13–16]. Engagement activities such as comments, likes, and shares are open-access engagement activities that can be accessed and controlled from social media postings [13, 14]. One of the well-known methods for analysing sentiment is sentiment analysis [17, 18]. Sentiment analysis, also referred as “opinion mining”, is an approach to natural language processing (NLP) that identifies the emotional tone behind a body of text. This is a popular way for organizations to determine and categorize opinions about a product, service, or idea [18–20].

So far, some studies have been conducted on sentiment analysis for social media content in order to analyse audience emotion [18, 21, 22]. Sentiment analysis with its ongoing improvement in mechanism, has been the best tool to measure online user’s emotions [23]. However, there were also limited studies on crisis monitoring using sentiment analysis. The techniques on how to detect pre-crisis from sentiment (user emotion) posted over social media are still lacking. Meanwhile, there has no in-depth analysis done to identify the pattern of negative sentiment or its intensity.

Therefore, this study aims to formulate pre-crisis detection formula by implementing sentiment analysis over extracted comments from selected Facebook postings. The pattern of sentiment and its intensity are two important elements which require further analysis before a formula for pre-crisis detection can be formulated [10, 11, 24]. Therefore, in this research, content framing on Facebook postings was used to evaluate the pattern of sentiment. The contents of postings, inclusive of emotion and reaction, trigger positive and negative polarity view, which will be measured using generic content framing theory [25] and the positive and negative attribute scale (PANAS) [26].

Finally, partial least squares structural equation modelling (PLS-SEM) will be employed to statistically measure the research model and hypotheses. The formula as formulated by the authors herein is to be expected to assist the Royal Malaysia Police as well as any monitoring body to predict criminal activities or crises from their organisation’s social media platform.

The findings of this study can assist organisations, policymakers, and governments to monitor and strategize their big data generated on social media. Employing sentiment analysis organisations can efficiently understand their audience, predict crises and prevent them. Organisations also can control the direction of news reporting content by implementing balanced generic framing on social media platforms to improve positive perspective on their audience feedback. The findings of this study aim not only to improve the public’s trust but also to avoid unnecessary creation of fake or false information posts in the future. Crisis can definitely be prevented if the organisation can manage the spread of negative sentiment wisely.

This paper consists of 5 sections namely Introduction (Section 1), Literature Review (Section 2), Methodology (Section 3), Result (Section 4) and Discussion (Section 5).

## Literature review

### Sentiment analysis for social media content

Sentiment analysis, otherwise known as “opinion mining” is the process of determining the emotional tone behind a series of words, used to gain an understanding of attitudes, opinions, and emotions expressed within an online text [18, 20]. Sentiment analysis tools are essential to detect and understand user feelings [18, 20]. Organisation use these tools to understand how their social media followers feel, which can be used to improve service delivery [18, 27, 28]. There are four types of sentiment analysis: fine-grained sentiment, emotion detection, aspect-based analysis, and intent analysis. Only fine-grained sentiment and emotion detection will be employed for this study, whereby fine-grained sentiment will categorized the comments into positive, negative, and neutral polarities while emotion detection sentiment will analysed emotions portrayed in each polarity [29, 30]. Aspect-based and intent analysis were deeper analysis where, for crisis detection, fine-grained sentiment and emotion detection were adequate to assist in pre-crisis detection according to crisis management theory [25, 31–36].

The emotion detection sentiment analysis was specifically designed and adopts the PANAS Scale [37, 38] to evaluate the user’s emotion towards the case study. After the sentiment polarity has been specified, the PANAS Scale, which has two categories: the Positive Polarity Subscale and the Negative Polarity Subscale, was used to assess only the user’s emotions [37, 38]. The positive polarity subscale consists of joy, interest, and activation subscale, while the negative polarity subscale consists of afraid and upset subscales [37, 38].

### Previous studies

Table 1 below shows the recent previous studies conducted on sentiment analysis for social media content to analyse audience’s emotion:

**Table 1:** Previous study summary.

Year	Scholar	Objective	Finding
2018	Paula Fortuna, Sérgio Nunes [21]	This study discusses the complexity of the concept of hate speech, defined in many platforms and contexts, and provides a unifying definition.	The development and systematization of shared resources, such as guidelines, annotated datasets in multiple languages, and algorithms, is a crucial gap in advancing the automatic detection of hate speech.
2020	Nikhil Kumar Singh, Deepak Singh Tomar & Arun Kumar Sangaiah [18]	Overview comprehensive sentiment analysis method to tackle issue on analysing comment in slag language that contains symbols, idioms, misspelled words sarcastic sentences. The improvement includes social media data which have curse of dimension problem i.e. high dimension nature of data that requires specific pre-processing and feature extraction, which leads to improve classification accuracy.	POS is most suitable feature extraction technique with SVM and Navie Bayes classifier. Whereas Random Forest and linear regression provides better result with Hass tagging.
2020	Manon Metz Sanne Kruikemeier Sophie Lecheler [22]	Investigates both the usage and consequences of self-personalization on Facebook.	The study shows that the use of a more emotional and private style provides a beneficial tool for politicians’ impression management. Publishing emotional and private content yields positive effects on audience engagement, suggesting audiences’ demand for more intimate and emotional impressions of public figures on the web.
2021	Antoine Banks Ernesto Calvo David Karol Shibley Telhami [39]	Assess the direct and unconditioned effect of exposure to tweets on perceived ideological polarization of candidates and parties using framing.	Social media frames increase contrast effects between voters and candidates.

Referring to the summary of previous studies from Table 1 above, we can conclude that sentiment analysis with its ongoing improvement on its mechanism, has been the best tool to measure online user’s emotion, but the techniques on how to detect pre-crisis from sentiment (user’s emotion) posted on social media are still lacking. Therefore, we explore framing theory to reveal and identify its role on sentiment buildup, as framing is one of several approaches in measuring user’s reaction in social media and further identify on how we can predict crisis from the sentiment build up.

### Generic framing theory (Underpinning theory)

In this study, sentiment was study from the comment where the formation of public opinion builds up. Even exploring the idea behind the contents of postings is crucial [33, 40], the formation of public opinion in modern paradigm (online comments/feedbacks) towards postings created on social media were not much explored especially for the purpose of crisis detection. The direction of news reporting also known as framing will drives on how audience thinks and act (response) [33, 40]. Comment (response) is one the public opinion form where this response from the audience were influence base on news contents (postings) [33, 40]. Therefore, the crucial part to study sentiment is not only comments but also postings where the source of comments come from. Framing is the best theory to be explored further to measure sentiments as this theory evaluate the direction of news as well as maps the ideology of agenda setting [25, 31–35, 41].

Previously scholars had utilized the generic content framing theory as in to map the news reporting agenda on television and newspaper [25, 31, 35, 36, 41]. Brugman and Burgers (2018) for example, had conducted a systematic review on the use of these frame types in the 21^st^-century political-framing experiments to establish whether and how scholars’ positions in these debates have changed across disciplines and over time. There were also conceptualized and measure [33, 40] on framing done by outlining an integrated process model of framing that includes production, content, and media use perspectives [25, 31, 35, 36, 41]. Vreese [32] concludes with an identification of contentious issues in current framing research.

Semetko and Valkenburg [25, 42] extended their findings on prior studies to look at the prevalence of the framing characteristic. One of their findings reveals that responsibility and conflict frame were the dominant contents for serious types of news while human interest frame was found to be dominant content for sensationalist types of news. For crisis detection we would like to recheck whether the direction of news reporting found by Semetko and Valkenburg [25] is reliable to be practised for crisis detection and to be contributed towards sentiment build up. None of the recent research has discussed about framing contribution for crisis detection. Most of them merely utilize the five (5) frame attributes by Semetko and Valkenburg [25, 42] to identify dominant frames [31, 34, 36]. One of the studies examines the global media framing of coronavirus disease 2019 (COVID-19) to understand the dominant frames and how choice of words compares in the media [34]. They conclude that global media coverage of COVID-19 was high, but the framing lacks coherence and sufficient self-efficacy and this can be associated with media’s obsession for breaking news [34].

The dominance of these frames not only impacts public perceptions and attitudes toward the pandemic, but it also puts people with pre-existing health disorders at danger of worsening their symptoms owing to dread or panic attacks [34]. This approach relates on how the content framing contributes towards fear and panic attacks [34]. However, whether the evaluation can best describe when the crisis (fear and panic attacks) started has not been revealed.

**Fig 1:**
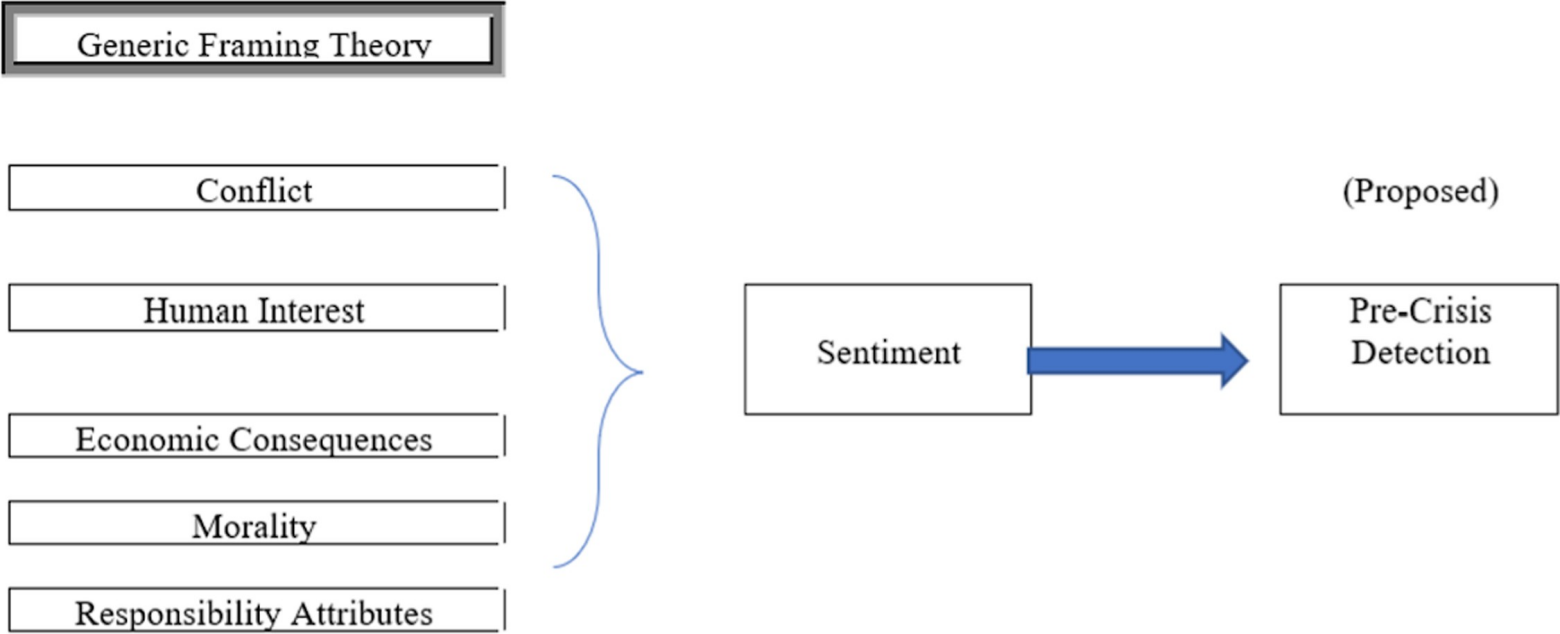
Theoretical framework. (A) Generic Framing Theory (B) Conflict (C) Human Interest (D) Economic Consequences (E) Morality (F) Responsibility Attributes (G) Sentiment (F) Pre-Crisis Detection.

Therefore, in this study, by referring to Fig 1, generic content framing as independent variables were designed in research framework to evaluate how the contents on posting influence and correlate towards sentiment formation amongst the community over social media. Our sample in this study (extracted Facebook posting) contains all the criteria in generic content framing (conflict, human interest, economic consequences, morality, and responsibility attribute). However, whether all these elements can best describe crisis detection remains unknown. Therefore, in this research, we would like to identify on which element in the generic content framing theory had correlation towards sentiment formation. Positive polarity subscale and negative polarity subscale were further divided into five (5) framing attributes [25, 42] in order to tie the news content postings on the case study. Generic frames are frames that are applicable to multiple issues [25, 31] and therefore is being selected for this study as the theory. The result therein is expected to assist in creating the sentiment pattern to further accommodate the pre-crisis detection formula.

Framing attributes used were conflict frame, economic consequences frame, human interest frame, morality frame and responsibility attributes frame [25, 34, 43] as a way of attracting audience interest, the conflict frame on the other hand highlights conflict between individuals, groups, or institutions. [44, 45].

Generic content framing was proposed to be implement for pre-crisis detection in this study because of its comprehensive coverage on variety of issue [25, 34, 43]. The attribution of responsibility frame for example was found as the most commonly used in the news, followed by the conflict, economic consequences, human interest, and morality frames, respectively [25, 32, 34]. The use of news frames depended on both the type of outlet and the type of topic [25]. Most significant differences were not between media but between sensationalist versus serious types of news outlets [25]. Independent media and conventional media more often used the responsibility and conflict frames in the presentation of news, whereas sensationalist outlets such as public figure more often used the human-interest frame [25, 32, 34]. Therefore, the generic content framing theory is expected to have the capability to be implemented for pre-crisis detection for various issues and is not limited to the case study. The first hypothesis (H1) was constructed as below:

H1: Conflict frame on Facebook postings is significantly influence public emotion and reaction on news reporting on sensitive issue.

Human interest frame brings a human face or an emotional angle to the presentation of a certain event, issue or problem as the human impact frame next to conflict and found to be common frame in the news [44, 45]. This frame also denotes an attempt to personalise, exaggerate, or emotionalize the news in order to pique and maintain listener’s attention [44, 45]. According to a study conducted by Park et al. [46] on alternative measures of infotainment’s effects on audience perception and reception of news on social media, focusing on infotainment coverage of North Korea. The findings show that human interest frame was one of the effective in frame in drawing public attention to news coverage [32, 34, 41]. Therefore, the second hypothesis (H2) was constructed as below:

H2: Human interest frame on Facebook postings is significantly influence public emotion and reaction on news reporting on sensitive issue.

Economic consequences frame reports and events, problem or issue in term of the consequences it will have economically on an individual, group, institution, region, or country [44, 45]. Previous study that focuses on the power of economic frames in shaping public perceptions found that economic frames affect how people think about the issue raise. The findings also assist the policymaking on action to be taken [44, 45]. Therefore, the third hypothesis (H3) was constructed as below:

H3: Economic consequences frame on Facebook postings is significantly influence public emotion and reaction on news reporting on sensitive issue.

Morality frame puts the event, problem or issue in the context of religious, tenets or moral prescriptions [44, 45] while responsibility attribute frame presents an issue or problem in such a way as to attribute responsibility for its cause or solution to either the government or to an individual or group [44, 45]. One of the previous studies conducted by [47], investigates cross-platform differences in social media by analyzing the contending candidates (Donald Trump and Hillary Clinton) who represent different political ideology during the 2016 presidential election. One of the findings found that for both Trump and Clinton followers on Twitter, conflict and morality frames consistently attracted retweeting behaviours while the responsibility frame seemed relatively less effective for audience engagement on social media. One possible explanation for this is that responsibility attribution requires a contextual understanding of an issue, which may be too cognitively effortful to process through a click-based engagement. This study in line with finding on manipulation of responsibility attributes was found nonsignificant and resulted in a reportedly weak manipulation in the study that aims to grow our current understanding of situational crisis communication theory by expanding on the conceptualization of causal responsibility as the primary mechanism contributing to the cognitive formulation of blame by stakeholder groups [48]. However, this study proposed responsibility attributes framing which deliver the understanding of an issue with contextual information had the potential to influence audience emotion and behaviour. Therefore, the fourth and fifth hypotheses (H4&H5) were constructed as below:

H4: Morality frame on Facebook postings is significantly influence public emotion and reaction on news reporting on sensitive issue.

H5: Responsibility attribute frame on Facebook postings is significantly influence public emotion and reaction on news reporting on sensitive issue.

## Methodology

Sentiment analysis was performed in this study using comments extracted from Facebook. According to statistic analyzer provider such as Socialbakers, Statisca, Digital Business Lab and many more, Facebook is the number one social media platform used by Malaysian online user comprise of significant majority of 80–90% Malaysian online population. Thus, Facebook has been selected as preferred social media platform in this study. To study the emotion towards news reporting on Facebook, selected postings were extracted. Posting extraction steps starts with keyword search on Facebook search column. The only keyword that we used to best describe the case study is ‘Kuil Mariamman’ (Mariamman Temple). The postings were selected and comments from the postings were extracted. The posting selected had met at least one of these criteria [24, 27, 28, 49]:

- Listed among top 10 Facebook page in Malaysia (SocialBakers website) and/or
- Consist of top post through keyword search
- Monitoring body Facebook page (Royal Malaysia Police)

According to [50], the population of Malaysia is approximately 30 million up to date, the sample size should be not less than 384 to best describe the population [50, 51]. We had limited the top ten (10) Facebook Page to ensure that the Facebook selection contains high follower to get appropriate exposure for each posting selected on particular Facebook page. The second criteria were set to ensure the posting had reach to more social media population. Therefore, only the top posting (posting with high engagement rate) was selected. The third criteria were set as the benchmark on news reporting content. Monitoring body referred to are the relevant organisation which is responsible towards the crisis and for this case study is the Royal Malaysia Police. Therefore, official Royal Malaysia Police Facebook page will be one of the Facebook page selected for this study.

From the screening process above, there were total of 56 posting shortlisted out of thousands existing posting derive from 10 Facebook pages (Table 2). As shown in Table 2, the postings were then grouped into four (4) agenda settings consisting of monitoring body–Royal Malaysia Police, conventional media—Berita Harian, Harian Metro & Sinar Harian, public opinion leader—Najib Razak (Former Prime Minister), Muhyiddin Yassin (Home Minister during the crisis) & Mahathir Mohamed (Prime Minister during the crisis) and independent portal (Siakap Keli, MalaysiaKini BM Version & Rotikaya) [11, 52–54]. The four (4) agenda settings derives from three (3) agenda settings proposed from the previous study on agenda setting in social media sphere [52]. There were organisational agenda (monitoring body and conventional media), individual agenda (public opinion leader) and public agenda (independent media). Organisational agenda was divided into two (2) elements namely monitoring body and conventional media to evaluate the organisational agenda component individually.

**Table 2:** Posting distribution according to agenda setting.

No.	Sources	Facebook Page	*n* Posting	*n* Comment
1	Monitoring Body	Polis Diraja Malaysia (Royal Malaysia Police)	10	2,743
2	Mainstream Media	Berita Harian	10	24,499
		Harian Metro	10	26,927
		Sinar Harian	9	14,523
3	Public Opinion Leader	Najib Razak (Former Prime Minister)	1	5,900
		Muhyiddin Yassin (Current Prime Minister)	3	864
		Dr. Mahathir Mohamed (Former Prime Minister)	2	2,897
4	Independent Media	Siakap Keli	7	18,482
		MalaysiaKini (BM Version)	3	1,052
		OHBULAN!	1	164
Total			56	98,051

The unit of analysis for this study is the Facebook postings and comments. Facebook platform was selected for this study due to its rank number 1 open-source social media platform in the most of the world including Malaysia according to a lot statistical analyser such as Social Baker, Statisca, Digital Business Lab and many more. According to Digital Business Lab statistics retrieved from https://digital-business-lab.com/2022/07/%E2%91%A1-social-media-penetration-in-malaysia-research/#:∼:text=As%20of%20January%202022%2C%2089,users%20from%202021%20to%202022, users from Facebook platform contribute 88.7% of the social media population in Malaysia. Comments were extracted for the analysis as the comments is the form of public opinion in modern paradigm where it carries sentiments [18, 20]. There was a total of 98,051 comments extracted from 56 selected postings. Generic content framing was measure on the selected postings where the comments been extracted. The postings and comments distribution are as shown as per Table 2.

The time frame of the case study is between 26^th^ of November 2018 until 30^th^ of November 2018. The starting of the time frame was decided as of 26^th^ November as that is the date where the first posting had been identified. The crisis benchmark was on 27^th^ November 2018 at 1.00 am where the incident of riot occurred as the result of racial provocation over social media resulting lots of injuries, major damage, and loss of life. The 30^th^ November was marked as the date of the end of research time frame as that is the starting date where number of posting decreased as well as engagement activity for each posting were also decreased. News postings were taken from monitoring body, mainstream media, public opinion leader and independent media while comments made on the postings were categorised into sentiment polarity positive, negative and neutral respectively. Framing was used as the theoretical framework. Five attributes of framing conflict frame, economic consequences frame, human interest frame, morality frame, responsibility attributes as independent variables adopted from [25] while emotion and reaction (sentiment analysis) using Positive and Negative Affect Scale PANAS [37, 38] as dependent variable as per Table 2.

Sentiment analysis using Application Programme Interface (API) webtools was performed to reveal the sentiment polarity distribution [24, 27, 55, 56]. All the comments from the selected Facebook’s posting were then run into the algorithm machine to examine the sentiment polarity. The sentiment analysis involves five (5) steps consist data extraction, data cleaning, data language detection, data translation and sentiment analysis. The first step is data extraction, the process of extracting comments is done by using a web-based comment exporter from the link https://www.commentexporter.com/. The selected Facebook post links were used to extract the comments including their timestamps. The data were then transferred to MYSQL web database for storage. This online storage enables researcher to access the data anywhere and at any time using any device.

The second step is the data cleaning process, the cleaning process involves the integration of MYSQL web and excel where this process involves removing redundant and incorrect values in data that is meant for analysis [17, 18]. The third step is language detection, while the fourth step is translation. In these 2 steps, the extracted comments were then identified for their language and translated using a web-based application developed to translate Malay language to English. The translation process uses the Google Neural Machine Translation (NMT) model. Finally, sentiment classification using Vader via the link https://github.com/cjhutto/vaderSentiment is used to perform sentiment analysis to classify the comments according to negative, neutral and positive polarity [24, 27, 55, 56].

Highly negative comment polarity distribution is detected from the comment’s distribution as per Table 3 herein. The posting was then analysed using content analysis method. The evaluation on emotion and reaction using PANAS scale with the sentiment polarity data through sentiment analysis were then analysed to reveal the correlation and influences between independent variables (conflict frame, economic consequences frame, human interest frame, morality frame and responsibility attributes frame) and dependent variables (emotion and reaction) using PANAS Scale.

**Table 3:** Sentiment polarity distribution.

Polarity	Total comment	Percentage
Negative	72473	83%
Neutral	6870	8%
Positive	7516	9%
Grand Total	86859	100.00%

In regards to data analysis, content analysis was first performed on the posting contents and comments to evaluate the sentiment pattern throughout the time frame for pre-crisis detection recognition descriptive analysis and then SEM was used to statistically analysed and measured the research model reliability as well as the correlation for each variable. The collection of data and analysis method complied with the terms and conditions of the data source.

## Result

### Sentiment and content analysis

Result from sentiment analysis towards 98,051 comments from 56 postings were shown in Table 2. There were 72,473 negative comments, 6,870 neutral comments, and 7,516 positive comments. According to the result, the total comments that available for sentiment analysis after cleaning process (step 2) was 86,859. Meaning that some of the comment was removed in online text cleaning, white space removal, expanding abbreviation, stemming, stop words removal, negation handling and finally feature selection [27, 28, 57]. This is to filter the irrelevant comments that does not carry sentiment in the contents to improve sentiment analysis result. This result was then analysed for content analysis using coding sheet with 43 indicators as shown in Table 4. The 43 indicators derive from all six (6) variables where dependent variables PANAS scale comprise of 20 indicators, conflict frame four (4) indicators, human interest frame five (5) indicators, economic consequences frame four (4) indicators, morality frame five (5) indicators and, responsibility attribute frame five (5) indicators.

**Table 4:** Indicator distribution according to composite.

Composite	Indicator	Definition	Reference
**Emotion & Reaction (PANAS)**	V1A1	Does the article made netizen feel distressed?	[37]
	V1A2*	Does the article made netizen feel afraid?	
	V1A3	Does the article made netizen feel nervous?	
	V1A4*	Does the article made netizen feel jittery?	
	V1A5*	Does the article made netizen feel scared?	
	V1B1	Does the article made netizen feel upset?	
	V1B2	Does the article made netizen feel guilty?	
	V1B3	Does the article made netizen feel hostile?	
	V1B4	Does the article made netizen feel irritated?	
	V1B5	Does the article made netizen feel ashamed?	
	V1C1*	Does the article made netizen feel excited?	
	V1C2*	Does the article made netizen feel proud?	
	V1C3*	Does the article made netizen feel enthusiastic?	
	V1D1	Does the article made netizen feel interested?	
	V1D2*	Does the article made netizen feel strong?	
	V1D3	Does the article made netizen feel determined?	
	V1E1	Does the article made netizen feel active?	
	V1E2	Does the article made netizen feel alert?	
	V1E3	Does the article made netizen feel attentive?	
	V1E4*	Does the article made netizen feel inspired?	
**Conflict Frame**	V3A1	The article/post reflects disagreement to the issue?	[25, 41]
	V3A2*	The article/post expressed the opposition point of view?	
	V3A3*	The post includes comment from the opposition?	
	V3A5	The article/post can cause conflict between individual, groups & institutions?	
**Human Interest Frame**	V3B2	The article/post emphasize how individuals and groups are affected by the riot?	[25, 41]
	V3B3	The article/post contain visual images that might trigger sympathy or compassion?	
	V3B6	The article highlights authorities helping the victims?	
	V3B7	The article/post portray sympathy to the victims?	
	V3B8	The article includes visual of individual helping victims?	
**Economic Consequences Frame**	V3C1	Financial losses or gains now or in the future were mentioned	[25, 41]
	V3C2	Value of loses from the riot were mentioned	
	V3C4	There is reference to economic consequences of pursuing or not pursuing a course of action	
	V3C5	The article/post mention the family of the affected victims affected financially	
**Morality Frame**	V3D1	There is/are any moral message?	[25, 41]
	V3D2	This article/post make references to any religious, value or morality?	
	V3D3	The article/post offer specific social prescriptions?	
	V3D4	The article/post bring goodness to the readers?	
	V3D5	The article/post include reminder from an interest group?	
**Responsibility Attributes Frame**	V3E1	There is/are blaming occur in the captions?	[25, 41]
	V3E2	The article/post suggest solution(s) to the problem?	
	V3E3	The article/post mention an individual/group/institution is responsible for the riot?	
	V3E4	The article/post suggest the level of the government is responsible for the riot?	
	V3E5	The article/post mention the government reactions/actions to the riot?	

*These indicators were not included in latent variables due to the multicollinearity criteria of PLS-SEM

**Fig 2:**
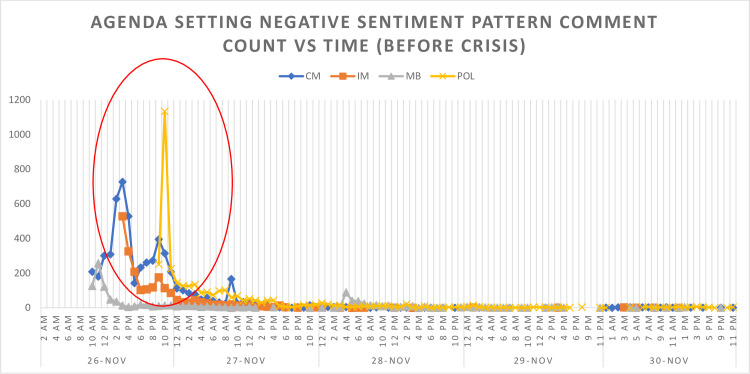
Negative comment intensity vs time for agenda setting (Before). (A) Agenda Setting Negative Sentiment Pattern Comment Count VS Time (Before Crisis) (B) Time on (C) Dates: 26 Nov; 27 Nov; 28 Nov: 29 Nov; 30 Nov.

According to Fig 2, the pre-crisis phase (intermittent high peak in negative comments) was able to trace from 10am to 12pm on the 26^th^ of November 2018.

### SEM-PLS

Data from content analysis were then compute to SMART PLS where value for composite A was calculated using Partial Least Square (PLS), value for composite B and structural model was obtained using calculation from bootstrapping. All 43 indicators measure for content analysis as per shown in Table 3 above were calculate for composite A, Composite Mode B and structural model.

#### Composite mode A

**Fig 3:**
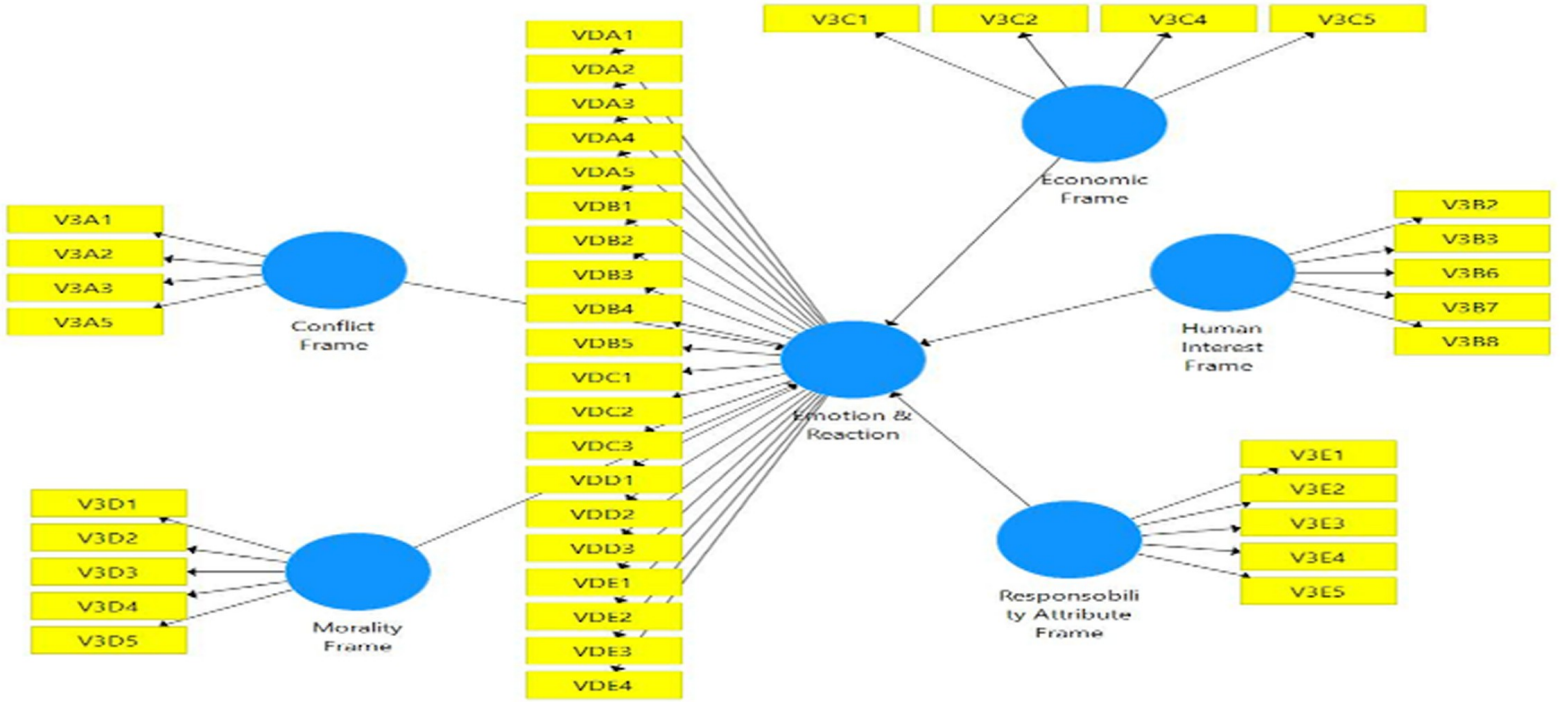
Research model. (A) Conflict Frame; Morality Frame (B) Emotion & Reaction (C) Economic Frame (D) Responsibility Attribute Frame (E) Human Interest Frame.

The composite measurement model in mode A was assessed in terms of individual item reliability, discriminant validity, convergent validity, and construct reliability, which were analysed using the through-loading factors shown in Fig 3. Composite reliability is more precise than internal consistency. It can be used in conjunction with PLS-SEM to handle a variety of loading indicators. Calculating convergent and discriminant validities was used to measure validity. The cut-off value of 0.7 is shown in Table 5 for three measurements: Dijkstra-rho Henseler’s coefficients, Cronbach’s alpha, and composite reliability. AVE value that is higher than 0.5 is adequate but if the Composite Reliability value is higher than 0.7, the low value of AVE is still acceptable [58] and therefore the third convergent validity is proven. Table 5 shows that the measurement model fits the criteria. Table 6 presents the results of discriminant validity through the Heterotrait-Monotrait (HTMT) correlation ratio. Because the confidence interval does not contain a zero value, all constructions comply with discriminant validity. Each variable is distinct from the others in this way. Table 7 shows the results in the measurement model above demonstrates that the construct is dependable and valid. The loading factors 0.70 or higher is preferred but as this study is exploratory research, the value of 0.4 and higher is still acceptable [59]. Finally, Table 8 shows the hypotheses results. According to this table, Economic Consequences Frame has significant influence on Sentiment Analysis (Emotion & Reaction) and Responsibility Attribute Frame has significant influence on Sentiment Analysis (Emotion & Reaction).

**Table 5:** Model testing result.

	Cronbach’s Alpha	rho_A	Composite Reliability	Average Variance Extracted (AVE)
Sentiment Analysis (Emotion and Reaction)	**0.893**	**0.920**	**0.910**	***0*.*362***

**Table 6:** Direct effect result.

HTMT Inference	Original sample	Sample mean	2.5%	97.5%
Conflict Frame → Sentiment Analysis (Emotion & Reaction)	0.030	0.068	-0.263	0.417
Economic Consequences Frame → Sentiment Analysis (Emotion & Reaction)	0.278	0.265	0.016	0.527
Human Interest Frame → Sentiment Analysis (Emotion & Reaction)	0.024	0.029	-0.314	0.408
Morality Frame → Sentiment Analysis (Emotion & Reaction)	-0.001	0.019	-0.291	0.297
Responsibility Attribute Frame → Sentiment Analysis (Emotion & Reaction)	0.425	0.429	0.045	0.816

**Table 7:** Significance of weight.

	Original Sample (Weight)	Loading	T	P Value	2.5%	97.5%
**V3A1 <- Conflict Frame**	0.706	**0.706**	3.570	**0.000**	0.147	0.908
**V3A2 <- Conflict Frame**	0.759	**0.759**	4.707	**0.000**	0.313	0.919
**V3A3 <- Conflict Frame**	0.454	**0.454**	1.667	***0*.*096***	-0.230	0.827
**V3A5 <- Conflict Frame**	0.680	**0.680**	3.584	**0.000**	0.170	0.898
**V3B2 <- Human Interest Frame**	0.793	**0.793**	9.294	**0.000**	0.580	0.904
**V3B3 <- Human Interest Frame**	0.828	**0.828**	11.335	**0.000**	0.648	0.929
**V3B6 <- Human Interest Frame**	0.875	**0.875**	26.275	**0.000**	0.800	0.925
**V3B7 <- Human Interest Frame**	0.859	**0.859**	17.261	**0.000**	0.744	0.934
**V3B8 <- Human Interest Frame**	0.624	**0.624**	5.244	**0.000**	0.346	0.807
**V3C1 <- Economic Frame**	0.911	**0.911**	32.671	**0.000**	0.844	0.952
**V3C2 <- Economic Frame**	0.872	**0.872**	24.895	**0.000**	0.796	0.933
**V3C4 <- Economic Frame**	0.762	**0.762**	9.478	**0.000**	0.566	0.882
**V3C5 <- Economic Frame**	0.811	**0.811**	11.789	**0.000**	0.644	0.908
**V3D1 <- Morality Frame**	0.800	**0.800**	6.146	**0.000**	0.454	0.916
**V3D2 <- Morality Frame**	0.660	**0.660**	4.636	**0.000**	0.318	0.861
**V3D3 <- Morality Frame**	0.821	**0.821**	8.038	**0.000**	0.594	0.928
**V3D4 <- Morality Frame**	0.796	**0.796**	5.674	**0.000**	0.443	0.924
**V3D5 <- Morality Frame**	0.840	**0.840**	6.171	**0.000**	0.490	0.943
**V3E1 <- Responsobility**	0.600	**0.600**	4.028	**0.000**	0.233	0.810
**V3E2 <- Responsobility**	0.683	**0.683**	6.931	**0.000**	0.457	0.831
**V3E3 <- Responsobility**	0.753	**0.753**	9.093	**0.000**	0.553	0.876
**V3E4 <- Responsobility**	0.887	**0.887**	26.891	**0.000**	0.812	0.938
**V3E5 <- Responsobility**	0.681	**0.681**	6.268	**0.000**	0.413	0.835
**VDA1 <- Emotion & Reaction**	0.492	**0.492**	3.599	**0.000**	0.184	0.711
**VDA2 <- Emotion & Reaction**	0.625	**0.625**	5.107	**0.000**	0.321	0.802
**VDA3 <- Emotion & Reaction**	0.605	**0.605**	5.501	**0.000**	0.344	0.772
**VDA4 <- Emotion & Reaction**	0.441	**0.441**	2.959	**0.003**	0.105	0.687
**VDA5 <- Emotion & Reaction**	0.706	**0.706**	7.791	**0.000**	0.488	0.838
**VDB1 <- Emotion & Reaction**	0.312	***0*.*312***	2.006	**0.045**	-0.032	0.575
**VDB2 <- Emotion & Reaction**	0.399	***0*.*399***	2.731	**0.006**	0.063	0.637
**VDB3 <- Emotion & Reaction**	0.205	***0*.*205***	1.220	***0*.*222***	-0.141	0.511
**VDB4 <- Emotion & Reaction**	0.095	***0*.*095***	0.610	***0*.*542***	-0.221	0.385
**VDB5 <- Emotion & Reaction**	0.366	***0*.*366***	2.473	**0.013**	0.043	0.619
**VDC1 <- Emotion & Reaction**	0.661	**0.661**	7.865	**0.000**	0.455	0.785
**VDC2 <- Emotion & Reaction**	0.579	**0.579**	5.518	**0.000**	0.335	0.746
**VDC3 <- Emotion & Reaction**	0.658	**0.658**	7.177	**0.000**	0.437	0.796
**VDD1 <- Emotion & Reaction**	0.807	**0.807**	10.561	**0.000**	0.623	0.914
**VDD2 <- Emotion & Reaction**	0.752	**0.752**	7.372	**0.000**	0.505	0.895
**VDD3 <- Emotion & Reaction**	0.827	**0.827**	16.551	**0.000**	0.710	0.899
**VDE1 <- Emotion & Reaction**	0.822	**0.822**	10.701	**0.000**	0.637	0.930
**VDE2 <- Emotion & Reaction**	0.674	**0.674**	7.215	**0.000**	0.463	0.829
**VDE3 <- Emotion & Reaction**	0.613	**0.613**	4.778	**0.000**	0.337	0.833
**VDE4 <- Emotion & Reaction**	0.708	**0.708**	7.836	**0.000**	0.496	0.847

**Table 8:** Whole sample result.

	Path	t	p	2.5%	97.5%
Direct effect					
Conflict Frame → Sentiment Analysis (Emotion & Reaction)	0.030	0.169	***0*.*866***	-0.263	0.417
Economic Consequences Frame → Sentiment Analysis (Emotion & Reaction)	0.278	2.156	**0.031**	0.016	0.527
Human Interest Frame → Sentiment Analysis (Emotion & Reaction)	0.024	0.132	***0*.*895***	-0.314	0.408
Morality Frame → Sentiment Analysis (Emotion & Reaction)	-0.001	0.007	***0*.*995***	-0.291	0.297
Responsibility Attribute Frame → Sentiment Analysis (Emotion & Reaction)	0.425	2.206	**0.027**	0.045	0.816
		**R**^**2**^ **= 0.448, Q**^**2**^ **= 0.12**			

#### Composite mode B

The collinearity between the indicators, significance, and relevance of the external weights were evaluated in the composite measurement model in mode B. To begin, the indicator was removed when the value of the impact factor variance (VIF = 3) was exceeded. As a result of this process, only the indicators shown in Table 3 above are not collinear. Second, the relevance of weights was analysed, as shown in Fig 3 above, with the relevance of indicators in construction for latent variables. Finally, 10,000 subsamples were used to begin bootstrapping and test the capability to detect outside weights that differed significantly from zero. Factor analysis show that non indicator had loading value less than 0.4 and shall be removed as shown in Table 4 above. Therefore, all indicators with loading value >0.4 retained to perform perfect research model.

#### Structural model

After verifying the appropriate values of the construction measurements, an assessment of the structural model was carried out using 10,000 resampling bootstraps. The path coefficients and the significance level of their 10,000 resampling bootstraps are reported in Table 7 and Fig 3. Furthermore, Table 7 also shows that this study also assesses quality by examining whether the predictive relevance of the whole model has a *Q*^2^-value above zero; therefore, it fits in the model predictions. The coefficient of determination (*R*^2^) also exceeds 0.1 for endogenous latent variables. As a result, the construct’s predictive power is satisfactory. Table 7 shows that the sentiment analysis (emotion and reaction) had correlation towards the economic consequences frame and responsibility attributes frame where the *p* values is < 0.05. Each variable also has a positive relationship with sentiment (emotion and reaction) and can be seen from the value of t-statistic which are all positive in Table 7. This model explains that the conflict frame, economic consequences, human interest, morality and responsibility attribute influence 44.8% of emotion and reaction (sentiment).

## Discussion

The postings and comments were examined using coding sheet and analysed further with Smart-PLS. There was a total of 86,859 comments analysed from 56 posting after the cleaning steps that derived from agenda setting which were three (3) online conventional media, two (2) monitoring body, four (4) online independent portal, and three (3) public opinion leader that met the criteria for posting and Facebook page selection. Based on the result of the bootstrapping test in Table 8, the following model of equation is obtained:

Y=0.278V3C+0.425V3E

Where,

Y: Pre-crisis detection(time)

V3C: Economic consequences(n negative comments)

V3E: Responsibility attributes (n negative comments)

The coefficient of conflict frame (V3A) 0.030, economic consequence frame (V3C) 0.278, human interest frame (V3B) 0.024 and responsibility attribute (V3E) 0.425 which are all positive whole morality (V3D) -0.001 is negative. This value can be interpreted as conflict frame, economic consequences frame, human interest frame and responsibility attributes positively influences sentiment (emotion and reaction) while morality frame negatively influenced. However only economic consequences frame and responsibility attribute frame are significant at *p* < 0.05 and therefore we conclude that even all variables had influenced the sentiment (emotion and reaction), but only economic consequences frame and responsibility attributes had correlation with sentiment (emotion and reaction). As these framing contributes to high negative comment according to Table 4, therefore, agenda setting (media) should enhance their direction of news reporting towards economic consequences and responsibility attributes frame to give a better impact towards emotion and reaction positively. One of the previous findings reveals that responsibility and conflict frame were the dominant content for serious types of news whilst human interest frame was found to be dominant content for sensationalist types of news [25]. Therefore, we conclude that:

H1: Conflict frame on Facebook postings is significantly correlate towards public emotion and reaction on news reporting on sensitive issue.

Conclusion: This study had identified conflict frame is the dominant types of content as it carry serious type of news however it does not influence the sentiment build up as all the agenda setting had delivered the conflict frame element in their news reporting very well to their audience [25].

H2: Human interest frame on Facebook postings is significantly correlate towards public emotion and reaction on news reporting on sensitive issue.

Conclusion: This study had identified human interest frame is the dominant types of content as it carry sensationalist type of news however it does not influence the sentiment build up as all the agenda setting had delivered the human-interest frame element in their news reporting very well to their audience.

H5: Responsibility attribute frame on Facebook postings is significantly correlate towards public emotion and reaction on news reporting on sensitive issue.

Conclusion: This study had identified the responsibility attribute frame is the dominant type of content because it also carries serious type of news similar to the conflict frame. This frame had been proven to have influenced the sentiment build-up as all the agenda setting had not delivered the responsibility attribute frame in their news reporting very well to their audience.

As per the result, H3 and H5 were accepted as economic consequences frame (H3) and responsibility attribute frames (H5) on news, and Facebook postings were significantly correlated towards public emotion and reaction on news reporting on sensitive issues. However, H1, H2, and H4 were rejected as conflict frame (H1), human interest frame (H2) and morality frame (H3) on news, and Facebook postings were not significantly correlate towards public emotion and reaction on news reporting on sensitive issues. Impact, particularly in framing, but not as a singular entity toward public sentiment. Public sentiment responded significantly to the economic consequences and responsibility attribute frame regardless of where or who posted the news on Kuil Mariamman Racial Riot. As referred from the previous study, Schmitz’s finding reveals that prejudice exists on multiple axes of hate and the Internet (for this case study, Facebook specifically) as a vehicle of hate [60] is strongly proven. Content from news reporting, especially framing on economic and responsibility attributes, could trigger prejudice (negative emotion) and hatred among social media users (community).

H3: Economic consequences frame on Facebook postings is significantly correlate towards public emotion and reaction on news reporting on sensitive issue.

Conclusion: Economic consequences frame is not the dominant content as not all the posting had covered this frame in their Facebook posting however it had been proved that it had significantly influence the sentiment build up and therefore this frame should be delivered consistently to the audience as the riot impact on economic is also one of the audience concerns. The finding reveals that economic consequences frame had to become the dominant content for serious and sensationalist type of news.

H4: Morality frame on Facebook postings is significantly correlate towards public emotion and reaction on news reporting on sensitive issue.

Conclusion: Morality frame is not the dominant content similar to economic consequences frame as not all the posting had covered this frame in their Facebook posting. As a result, morality frame also does not influence the sentiment build up. The finding reveals that morality frame can’t be prove to become the dominant content for serious and sensationalist type of news.

William and Burnap [5] computational criminology discovery, which renews our view of hate crime as a process rather than a single event for the digital age, is likewise in accordance with this research finding. The result shows that a process starting from offline action (crime) which involves the attack of Malay men in the temple to demolish the temple was what triggered the emotion of Malays and Indian nationwide. As emotion was triggered, more and more posting over social media has been posted to keep the Malaysian citizen updated. This process initiates variety of agenda settings that came up with news content through social media to map the social media opinion towards the issue. However, prejudice (sentiment) triggered due to sentiment provocation is much dominant than ability of reporting news to direct the reader’s rationality. As the issue becomes controversial, rise in engagement activities had steered up hate amongst the netizens, thus contributing towards high negativity which creates racial tensions then resulting in riot. The crime process therein can be prevented if there is intervention by the said monitoring body. However, such intervention can only be made possible if pre-crisis can earlier be detected with intensive handling including preparation at scene has been done as part of crisis management.

Result shown had indicated that conflict frame, human interest frame and morality frame had no significant correlation towards sentiment. This is the reason why the pre-crisis detection formula only utilize value from economic consequences frame as well as responsibility attribute frame. Pre-crisis formula can be improved if more posting which covers on all generic content framing is obtained. Therefore, to steer the community’s emotion in the desired direction, we recommend that the monitoring body, as well as agenda setting, to promote posting with contents that emphasises conflict between individuals, groups, or institutions as a means of capturing audience interest. Moreover, human face or an emotional angle may also be added towards a certain event, issue, or problem as part of human impact frame in order to personalise, dramatize, or emotionalize certain news so as to capture and retain audience interest [44, 45]. The same can also be put and categorised in the context of religion, tenets, or moral prescription [44, 45]. By striking a balance content which covers the said Generic Framing as a whole, monitoring body via their official Facebook page is able and is expected to gauge the community’s emotion and action whilst being able to response accordingly through crisis management plan in order to control harmful emotional instability that could lead to unwanted event such as riot.

According to Basic Econometric Study Books [50], result can be improved with the sample size increased. Given the circumstances that this research was conducted after two (2) years from the said incident, results from the Facebook search engine will also be affected. Current algorithm from Facebook had subsequently limits the data retrieval as part of their privacy measure. As such, search by keywords will show random posting which may or may not carry high engagement rate thus resulting in quality selective posting. This is due to posting which have higher number of engagement activity not appearing by keyword search.

In conclusion, this research has explored protentional framing theory so as to detect pre-crisis for the purpose of social media monitoring. There is no previous research that has employed generic content framing theory to measure sentiment for crisis prediction. Therefore, this study has found that by evaluating public sentiment (comments) towards generic content framing on Facebook posting, specifically on the economic consequences frame and responsibility attribute frame, we are able to detect and identify the pre-crisis phase before the crisis ever occurs (Fig 2), for which a formula for pre-crisis detection (Y = 0.278 V3C + 0.425 V3E) has also been created. However, this finding can be improved if the data can be collected during the said crisis period, where there will be a sufficient sample size to evaluate all types of framing. The monitoring body should improve their news reporting on their respective official Facebook page and by adhering to the required aspects in every framing, particularly on the economic effects and responsibility characteristics frames.
